# Design, Modeling and Simulation of a Liquid Jet Gyroscope Based on Electrochemical Transducers

**DOI:** 10.3390/mi12091008

**Published:** 2021-08-24

**Authors:** Dapeng Yang, Xiaohuan Wang, Junze Sun, Heng Chen, Chenhao Ju, Tingting Lin, Baofeng Tian, Fan Zheng

**Affiliations:** Key Laboratory of Geo-Exploration Instrumentation, Ministry of Education, College of Instrumentation and Electrical Engineering, Jilin University, Changchun 130012, China; ydp@jlu.edu.cn (D.Y.); wangxh19@mails.jlu.edu.cn (X.W.); sunjz19@mails.jlu.edu.cn (J.S.); chenheng20@mails.jlu.edu.cn (H.C.); juch6517@mails.jlu.edu.cn (C.J.); ttlin@jlu.edu.cn (T.L.); tianbf@jlu.edu.cn (B.T.)

**Keywords:** jet gyroscope, electrochemical transducer, liquid

## Abstract

We propose a novel liquid jet gyroscope based on electrochemical transducers, which uses electrolyte as the jet medium, and two electrochemical transducers placed symmetrically as the velocity measuring unit. The gyroscope includes a fluid pump to generate a jet flow, which flows into the jet chamber. Then, it is diverged into the shunt channels, pumped into reflux channels and merged by a fluid pump. The velocities of shunt flows are measured by two electrochemical transducers. The feasibility of the method was demonstrated in theory, and a 2D finite element model was built to simulate the dynamics of the liquid jet gyroscope. Simulation results confirm the effectiveness of the gyroscope, which has higher sensitivity in the near DC frequency band.

## 1. Introduction

Gyroscope is an important inertial sensing element to detect the angular rate of moving objects [1]. The fluid gyroscope was studied widely in its various forms due to its small size, low cost, high reliability, high shock resistance and high vibration resistance, with thermal convective gas gyroscope being the most representative [2,3]. It is especially suitable for use in the harsh environment on cars, tanks and shells. Since the thermal convective gyroscope uses gas as a jet medium, its sensitivity is limited because gas has a low density [4,5,6]. On this basis, some scholars put forward a novel liquid rate gyroscope using electro-conjugate fluid to improve sensitivity, which generates a powerful jet flow under the action of a high DC voltage [4]. However, the sensing principle of angular rate is still based on the universal gas rate sensor, which employs four hotwires placed in a constant temperature field to measure the deflection of jet flow. A local temperature field is formed around hotwires since they need to work in the heating state. The gas or liquid rate sensor makes the hotwires cool down gradually, and the output voltage changes under the action of jet flow, which will lead to measurement hysteresis.

The electrochemical transducer has the advantage of a high conversion factor with a very simple and suitable for mass production for the inertial measurement system. The sensors based on electrochemical transducer were used in seismology [7,8,9,10,11], navigation [12,13] and structural monitoring [14].

To overcome the shortcomings of low sensitivity, low conversion efficiency and long hysteresis time, a liquid jet gyroscope based on electrochemical transducers was investigated in this study. The fluid pump of the gyroscope generates a jet flow in the jet chamber, which is deflected by Coriolis force induced by angular rate. Then, the deflection of the jet flow is picked up by the electrochemical transducers installed in the shunt channels. Therefore, the gyroscope senses the angular rate by detecting the deflection of the jet flow. Compared to the conventional gas rate sensors, the electrochemical transducers could enhance several performances on the basis of existing fluid gyroscopes, such as simple structure, high conversion efficiency, high sensitivity and quick response.

In this paper, we designed a liquid jet gyroscope based on electrochemical transducers and introduced the principle of the gyroscope. The 2D finite element simulation model of the gyroscope was established, the simulation results analyzed and future research direction pointed out.

## 2. Theory and Mathematical Model

### 2.1. Electrochemical Transducer

**Figure 1:**
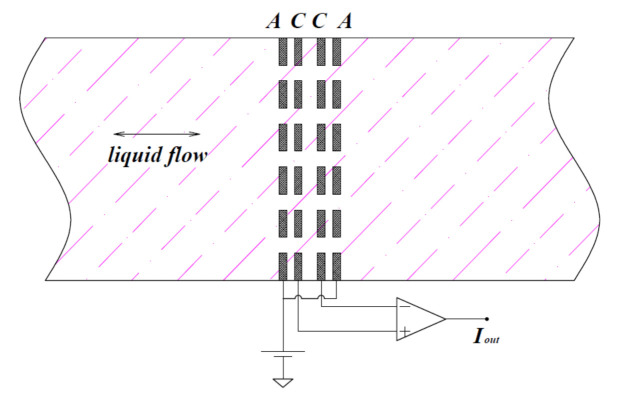
Schematic diagram of the electrochemical transducer. Anode/cathode/cathode/anode (ACCA) represents the anode-cathode-cathode-anode.

The schematic diagram of the electrochemical transducer is shown in Figure 1. The sensing element consists of four porous inert metal electrodes, normally arranged in anode/cathode/cathode/anode (ACCA) along the conduit [15,16,17,18,19]. Four electrodes are placed in one channel filled with electrolyte under the working condition, with each electrode isolated by a porous insulating film. A DC voltage is applied to the anodes via an external circuit, and the cathodes are the current measuring terminals. After electrifying and resting for some time, the electrochemical reaction between cathodes and anodes tends to balance, the ion concentration gradient near the two cathodes distributes symmetrically, and the current difference between the two cathodes is 0. When the electrolyte is driven to move relative to electrodes, the ions in the electrolyte will migrate, and the ion concentration gradient near the two cathodes will no longer distribute symmetrically. Therefore, the current in relation to the velocity of the electrolyte will be detected on the cathodes. The velocity of the electrolyte is expressed in the form of the current difference between the two cathodes.

The electrolyte used in this study is a mixed solution of *I*_2_ and *KI*, whereby the reversible electrochemical reaction $I_{2}+I^{-}⇌I_{3}^{-}$ occurs. Under the action of external voltage, the reaction on the surface of the cathodes and anodes are:

Cathodes:$\;I_{3}^{-}+2e\to 3I^{-}$

Anodes:$\;3I^{-}\to I_{3}^{-}+2e$

The currents on the electrodes are calculated with the expression:

(1) $$I_{out}=Fz\oint \left(\vec{N},\vec{n}\right)d\vec{S}.$$

Here, $F=9.648534\times 10^{4}\;C/\mathrm{mol}$ is the Faraday Constant for the active ions, z denotes the charge amount, $\vec{N}$ denotes the flux of the active ions in the electrolyte, $\vec{n}$ denotes the unit vector of a normal to the surface of an electrode and $\vec{S}$ denotes the surface area of the electrode.

### 2.2. Configuration of Gyroscope

**Figure 2:**
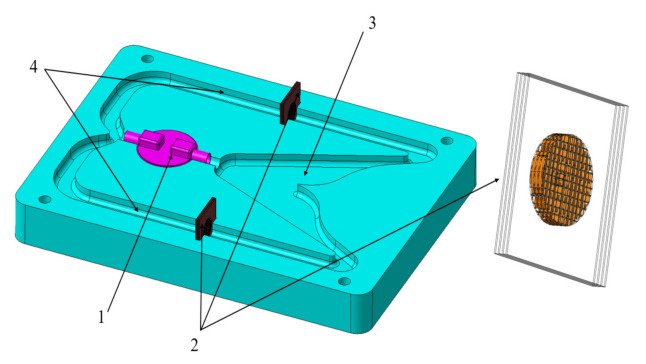
Configuration of the liquid jet gyroscope: 1. fluid pump; 2. electrochemical transducers; 3. jet chamber; 4. shunt channels.

As shown in Figure 2, the configuration of the electrochemical gyroscope comprises three parts: the gyroscope frame, the fluid pump and the electrochemical transducers. The gyroscope frame consists of two grooved walls, which form the closed chamber and channels. The electrolyte fills the chamber and channel and is driven by a fluid pump for circulating around the chamber and channels. After entering the jet chamber, the electrolyte stream would be split into two separate tributaries by the nose between shunt channels. Two electrochemical transducers are installed in the shunt channels for measuring the velocities of each channel. In the absence of rotation, the flow rates of electrolytes are symmetrical in each channel. When an external angular velocity occurs, the velocity direction of the electrolyte stream will be deflected due to the effect of Coriolis force, which will result in an unsymmetrical rate in each channel. The angular velocity acting on the gyroscope is thus reflected by the difference of the two electrochemical transducer outputs.

### 2.3. Mathematical Model

**Figure 3:**
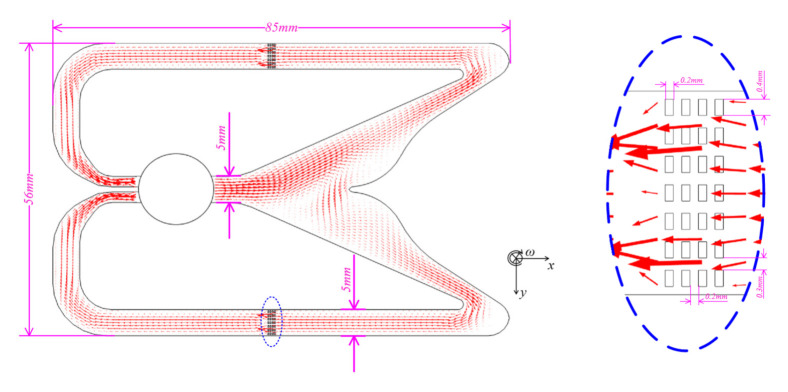
Schematic diagram of the liquid jet gyroscope. The jet flow velocity and direction are shown by the size and direction of red arrows.

The 2D mathematical model with the shape size of 56 mm × 85 mm is established based on the geometric structure shown in Figure 3, which also includes the key dimensions of the electrochemical transducers with the details of electrode thickness 0.2 mm, electrode aperture 0.3 mm, inter-aperture wall 0.4 mm and inter-electrode gap 0.2 mm. According to the principle of the gyroscope, the coupling of the fluid field and the electrochemical field is required to analyze in this paper.

The fluid field is solved by incompressible Navier–Stokes equations:

(2) $$\nabla \cdot \vec{u}=0;$$

(3) $$\rho \frac{d\vec{u}}{dt}=-\nabla P+\mu \nabla^{2}\vec{u}+\rho \vec{a}.$$

where $\vec{u}$ is the velocity vector of the electrolyte, *t* is time, $\vec{a}$ is external acceleration, P is pressure, ρ is electrolyte density, μ is dynamic viscosity. The external acceleration is expressed by Equation (4).

(4) $$\vec{a}={\vec{f}}_{C}/\rho =2\vec{\omega }\times \vec{u}.$$

where$\;{\vec{f}}_{C}$ and $\vec{a}$ are the force and acceleration from the Coriolis effect, and $\vec{\omega }$ is angular rate. The electrolyte density and dynamic viscosity are calculated using Equations (5) and (6):

(5) $$\rho =\rho_{w}+\overset{k}{\underset{i=1}{\sum }}c_{i}\left[B_{1i}+B_{2i}\left(T-273.15\right)+B_{3i}c_{i}\right];$$

(6) $$\mu =\mu_{w}exp\left\{\overset{k}{\underset{i=1}{\sum }}c_{i}\left[A_{0i}+A_{1i}\left(T-273.15\right)+A_{2i}c_{i}+A_{3i}{\left(T-273.15\right)}^{2}\right]\right\}$$

where $\rho_{w}$ is water density, $c_{i}\;$is electrolyte mass fraction, T is the temperature in Kelvin, $\mu_{w}$ is water dynamic viscosity and $A_{ij}$ as well as $B_{ij}$ are the coefficients, which can be found in Table 1.1 from the literature [20] for different salts.

To solve the electrochemical field for ion transport, the Nernst–Plank equations with electro-neutrality assumptions are employed.

(7) $$\frac{\partial C_{k}}{\partial t}=z_{k}m_{k}F\nabla \cdot \left(C_{k}\nabla \varphi \right)+D_{k}\nabla^{2}C_{k}-\vec{u}\nabla C_{k};$$

(8) $$\underset{k}{\sum }z_{k}C_{k}=0$$

here, $C_{k}$ is concentration, $z_{k}$ is charge number, $F=9.648534\times 10^{4}\;C/\mathrm{mol}$ is the Faraday Constant, φ is electric potential, $D_{k}$ is diffusion coefficient, $m_{k}$ is ion mobility solved by the Nernst–Einstein equation m=D/RT, where $R=8.314\;J/\left(\mathrm{kg}/\mathrm{mol}\right)$ is the gas constant.

According to the literature [16] from Zhanyu Sun and V. M. Agafonov, the Butler–Volmer equation, namely the boundary condition, is expressed approximately by Equation (9):

(9) $$2\vec{n}\cdot {\vec{N}}_{I_{3}^{-}}=-\frac{2}{3}\vec{n}\cdot {\vec{N}}_{I^{-}}=-k_{a}2C_{I_{3}^{-}}e^{\frac{-\alpha nF}{RT}\left(U-\varphi -E_{0}\right)}+k_{c}C_{I^{-}}e^{\frac{\left(1-\alpha \right)nF}{RT}\left(U-\varphi -E_{0}\right)}$$

where $\vec{n}$ is the out-normal of the electrode surface, $\vec{N}\;$is the ion flux, $k_{a}$ and $k_{c}$ are the anode and cathode reaction constants, *n* is the number of electrons exchanged, α is the charge transfer coefficient, *U* is the imposed electric potential at the electrodes and $E_{0}$ is the equilibrium potential.

The ion flux can be denoted by:

(10) $$\vec{N}=\vec{u}C-D\nabla C-zmFC\nabla \varphi .$$

Combining Faraday’s law in Section 2.1, we conclude that the output in each shunt channel is:

(11) $$I_{Sch,out}=I_{Sch,u}-I_{Sch,d}=Fz_{I_{3}^{-}}\oint \left({\vec{N}}_{I_{3}^{-}},\vec{n}\right)d{\vec{S}}_{Sch,u}-Fz_{I_{3}^{-}}\oint \left({\vec{N}}_{I_{3}^{-}},\vec{n}\right)d{\vec{S}}_{Sch,d}$$

where $I_{Sch,\;u}$ and $I_{Sch,\;d}$ are the output electric current of upstream cathode and downstream cathode and $S_{Sch,\;u}$ and $S_{Sch,\;d}$ are the surface area of the upstream cathode and downstream cathode, respectively.

The output current of the gyroscope is:

(12) $$I_{gys,out}=I_{Sch,dif}$$

where $I_{Sch,\;dif}$ is the electric current difference of the electrochemical transducer outputs in each shunt channel.

The following parameters are used in the paper: The fluid rate at the fluid pump outlet ${\vec{u}}_{outlet}$ varies from 0 to 0.5 mm/sec, and the pressure P at the fluid pump inlet is zero. A Zero-flux boundary condition was applied at the electrode for potassium, and a no-slip boundary condition was set for all solid surfaces. At the dielectric surfaces, an electric insulation boundary condition is employed for the electric field. The temperature is set to 300 K. The ion concentrations of electrolyte are $C_{I^{-}}=3960\;\mathrm{mol}/m^{3}$ and $C_{I_{3}^{-}}=40\;\mathrm{mol}/m^{3}$. We set the ionic diffusion coefficient at $D_{I^{-}}=D_{K^{+}}=2.8\times 10^{-9}{\;m}^{2}/s\;$and $D_{I_{3}^{-}}=2\times 10^{-9}{\;m}^{2}/s$, the charge numbers are $Z_{I^{-}}=Z_{I_{3}^{-}}=-1$ and $Z_{K^{+}}=1$. The imposed electric potential at the anodes is 0.8 V, the charge transfer coefficient is α=0.5 and the equilibrium potential is $E_{0}=0.54\;V$. The electrolyte density is found to be $\rho_{\omega }=1.473\times 10^{3}\;\mathrm{kg}/m^{3}$ and the electrolyte dynamic viscosity is found to be$\;\mu_{\omega }=1.4\times 10^{-3}\;\mathrm{Pa}\cdot s$. The anode and cathode reaction constants are assumed to be$\;k_{a}=k_{c}=4\times 10^{-9}\;m^{2}/s$.

## 3. Solver Validation

In this paper, the transient-state results are studied due to the frequency characteristic of the external angular rate. The 2D finite-element multi-physics model was built by COMSOL Multiphysics software. The solver was validated by simulating the model in Zhanyu Sun and V. M. Agafonov’s [16] paper, and similar results were obtained once the solution has reached a steady state. We used the same solver to calculate the current 2D model. A boundary layer grid was employed for the purpose of the precise solution. Both the direct method (PARDISO) and the full coupling are used to solve the transient model. The calculation relative error is controlled under$\;5\times 10^{-4}$.

## 4. Result and Analysis

### 4.1. The Time Domain Response

**Figure 4:**
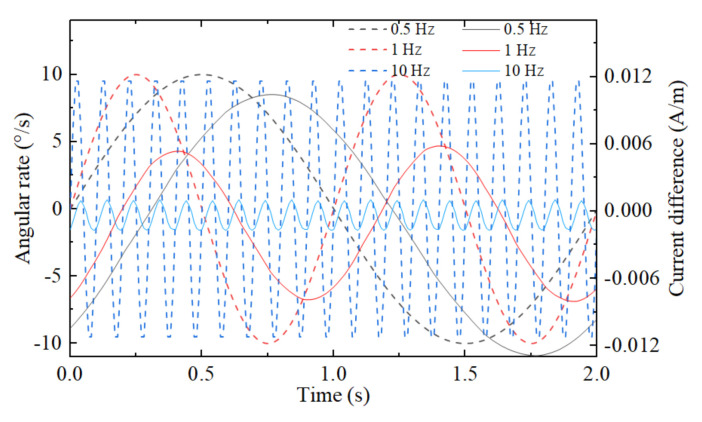
Angular rates and output current differences for the case of fre=0.5 Hz, fre=1 Hz and fre=10 Hz in the time domain. The dotted lines represent the relationship between external angular rate and time, and the solid lines represent the relationship between current difference and time.

In order to observe the output signals waveform, we simulated the concept model with the time-dependent solver at these parameters: the jet flow rate${\;\vec{u}}_{\mathrm{outlet}}=0.3\;\mathrm{mm}/s$, the amplitude of the input angular rate 10°/s, frequency 0.5 Hz, 1 Hz and 10 Hz, respectively. After the initial 30 s, the outputs of the gyroscope were considered to be steady-state results. As shown in Figure 4, the relationship between external angular vibration and time is represented by dotted lines corresponding to the *X*-axis and left *Y*-axis, and the relationship between output response and time is represented by solid lines corresponding to the *X*-axis and right *Y*-axis. It is proven that the angular rate can be sensed by a liquid jet gyroscope based on electrochemical transducers. The output of the gyroscope is the standard sinusoidal signal in the condition of the external standard sinusoidal angular vibration.

### 4.2. The Frequency Domain Response

**Figure 5:**
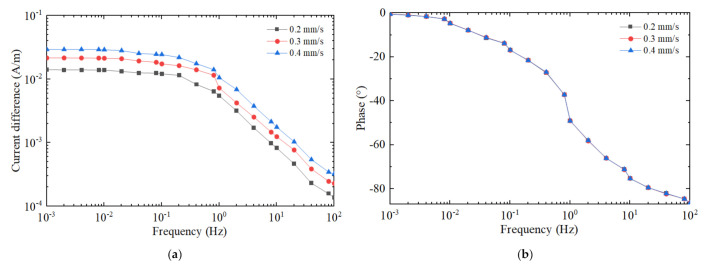
The output responses at different jet flow velocities in the frequency domain. The jet flow velocity is 0.2 mm/s, 0.3 mm/s and 0.4 mm/s, respectively. (**a**) Amplitude–frequency response. (**b**) Phase–frequency response.

Figure 5a shows the output response curves of amplitude versus frequency, and Figure 5b that of phase versus frequency. The amplitude of the input angular rate is set to 10°/s, the frequency ranges from 0.001 Hz to 100 Hz and the jet flow rate to 0.2 mm/s, 0.3 mm/s and 0.4 mm/s, respectively. The simulation results indicate that the output signal amplitude increases as the jet flow rate increases. However, the assumption of laminar flow will collapse if the flow rate is excessively high, which is not considered in this paper. The output signal amplitude curves are nearly flat when the frequency is lower than 0.1 Hz, yet attenuate in −20 db higher than 1 Hz. The output signal phases lag to 90° gradually with the increase in frequency, staying almost the same at different jet flow rates. The amplitudes and phases of the output signals vary with the frequency of the external angular vibration, which is a tricky problem when it comes to gyroscope application and therefore needs to be corrected by the external circuit.

**Figure 6:**
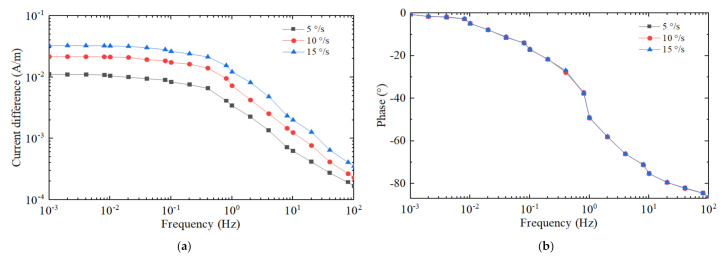
The output responses at different external angular rates in the frequency domain. The external angular rate is 5°, 10° and 15°, respectively. (**a**) Amplitude–frequency response. (**b**) Phase–frequency response.

At the jet flow rate 0.3 mm/s, the frequency 0.001 Hz to 100 Hz and the amplitude of angular rate 5°/s, 10°/s and 15°/s, we obtain the results shown in Figure 6 with coordinate axes similar to Figure 5. It can be observed that the output signal amplitude increases as the external angular rate increases in the range of 0.001–100 Hz. The output signal phases are almost the same at the different angular rates, as shown in Figure 6b.

**Figure 7:**
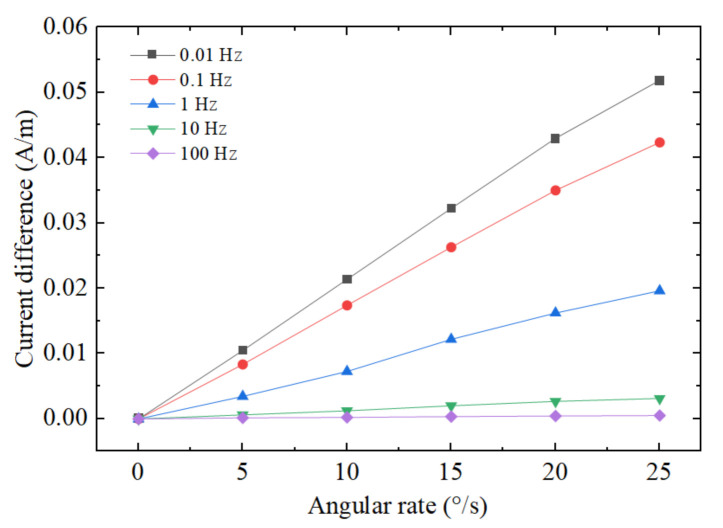
The output linearity curves at different frequencies.

Figure 7 shows the output linearity curves, i.e., the output current difference versus the amplitude of the external angular rate at different frequencies. All of the data presented in Figure 7 come from the simulation results, with a jet flow rate at 0.3 mm/s and the angular rate from 0°/s to 25°/s by a 5°/s step. As shown in Figure 7, the output current difference changes linearly with the angular velocity under the same frequency condition. Under the conditions of low frequency and strong angular rate, the linear relationship is destroyed due to the fact that the electrolyte flow exceeds the monotonous response zone for an electrochemical transducer.

## 5. Discussion

In order to estimate the performance of the electrochemical jet gyroscope in terms of small-signal resolution, we compare the gyroscope sensitivity calculated with the self-noise of the electrochemical transducer. According to the literature [21], the noise spectral density of the transducer output current:

(13) $$\langle I_{\omega }^{2}\rangle \sim S_{el}n^{2}/d^{2}.$$

where $S_{el}$ denotes the electrode surface area, n denotes the concentration of the electrolyte active substance and d denotes the inter-electrode gap.

We now assume that the 2D transducers are extended by 5 mm in the normal vector of a plane for fabricating the real prototype, which means $S_{el}=42{\;\mathrm{mm}}^{2}$. Combined with other parameters of n=0.04 mol/l and d=0.2 mm, referring to the Equation (5) in the literature [21], the noise spectral density of the prototype is expressed by the following:

(14) $$\langle I_{\omega }^{2}\rangle =\frac{5.0\times 10^{-18}}{{\left(1+\omega \right)}^{0.55}}A^{2}Hz.$$

**Figure 8:**
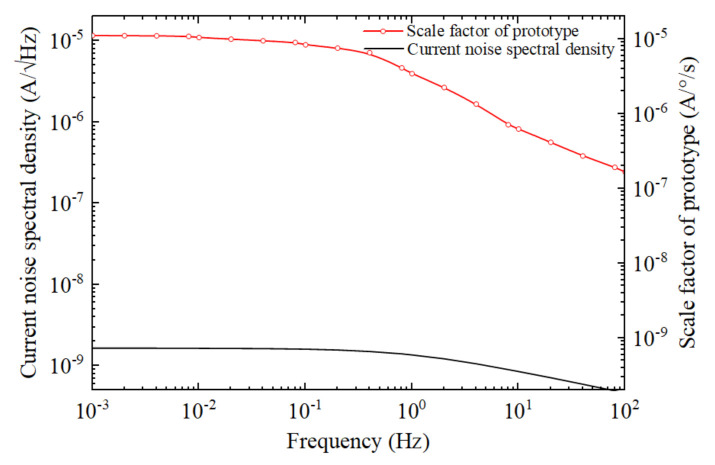
The scale factor and the current noise spectral density of the 5 mm-thickness prototypes versus frequency using the parameters of the jet flow rate 0.3 mm/s and angular rate 10°/s.

Moreover, the scale factor of the prototype should be the previous results multiplying by$\;5.0\times 10^{-3}\;m$. Figure 8 shows the scale factor and the current noise spectral density of the 5 mm-thickness prototypes versus frequency using the parameters of the jet flow rate 0.3 mm/s and angular rate 10°/s. The equivalent noise in angular velocity units can be obtained by calculating the current noise divided by a scale factor, and typical numerical values are$\;2.0\times 10^{-4}{}^{\circ}/s\;\mathrm{at}\;0.001\;\mathrm{Hz}$, $2.6\times 10^{-4}{}^{\circ}/s\;\mathrm{at}\;0.1\;\mathrm{Hz}$, $1.8\times 10^{-2}{}^{\circ}/s\;\mathrm{at}\;10\;\mathrm{Hz}$ and $3.7\times 10^{-2}{}^{\circ}/s\;\mathrm{at}\;100\;\mathrm{Hz}$.

Due to the above calculation results being only derived from the effect of Coriolis force and ignoring the effect of inertial force, the scale factor results should be more accurate in the low-frequency region and smaller than the real value with the increase in frequency, which causes the equivalent noise looks a little big in the high-frequency region.

The performance of the jet gyroscope should be improved by pushing the velocity appropriately on the premise of less than the saturation detecting velocity of the electrochemical transducer. Moreover, increasing the length of the jet chamber can receive more effect of Coriolis force on the fluid deflection, and reducing the inter-electrode gap of the electrochemical transducer can effectively improve the sensitivity of transducers.

## 6. Conclusions

We simulatively studied the working state of the liquid jet gyroscope based on electrochemical transducers under the action of Coriolis force. Different frequencies of external angular rate were employed to excite the gyroscope. The results in both time and frequency domains were analyzed in detail. In the time domain, the output of the gyroscope is the standard sinusoidal signal under the input of the external standard sinusoidal angular rate vibration. In the frequency domain, the scale factor of the gyroscope increased with the jet flow rate, markedly declined above 1 Hz input angular rate, there was a good linear relationship between input angular rate and gyroscope output, and also the output signal phases were basically the same at different input angular rates. The equivalent noise in angular velocity units can reach the level of $10^{-4}{}^{\circ}/s$ in the near DC frequency band. The performance of the jet gyroscope could be further improved by pushing the velocity, adjusting the size of the gyroscope and transducers. The feasibility of the method was analyzed theoretically, and the superiority of the method is verified by simulation. In future studies, we will focus on the combined action of Coriolis force and inertial force for the performance of electrochemical jet gyroscope.

## References

[1] Dau V.T., Dinh T.X., Tran C.D., Bui T.T., Phan H.T. (2018). A Study of Angular Rate Sensing by Corona Discharge Ion Wind. Sens. Actuat. A-Phys..

[2] Dau V.T., Dao D.V., Shiozawa T., Kumagai H., Sugiyama S. (2006). Development of a dual-axis thermal convective gas gyroscope. J. Micromech. Microeng..

[3] Dau V.T., Dao D.V., Shiozawa T., Kumagai H., Sugiyama S. (2005). A single-axis thermal convective gas gyroscope. Sens. Mater..

[4] Takemura K., Yokota S., Suzuki M., Edamura K., Kumagai H., Imamura T. (2009). A Liquid Rate Gyroscope Based on Electro-conjugate Fluid. Sens. Actuat. A-Phys..

[5] Dau V.T., Dinh T.X., Tran C.D., Bui P.N., Vien D.D., Phan H.T. (2018). Fluidic Mechanism for Dual-axis Gyroscope. Mech. Syst. Signal Pr..

[6] Dau V., Dinh T.X., Sugiyama S. (2009). A MEMS-based silicon micropump with intersecting channels and integrated hotwires. J. Micromech. Microeng..

[7] Agafonov V.M., Neeshpapa A.V., Shabalina A.S. (2015). Electrochemical seismometers of linear and angular motion. Enc. Earthq. Eng..

[8] Levchenko D.G., Kuzin I.P., Safonov M.V., Sychikov V.N., Ulomov I.V., Kholopov B.V. (2010). Experience in seismic signal recording using broadband electrochemical seismic sensors. Seism Inst..

[9] Bernauer F., Wassermann J., Igel H. (2012). Rotational sensors—A comparison of different sensor types. J. Seismol..

[10] Leugoud R., Kharlamov A. (2012). Second generation of a rotational electrochemical seismometer using magnetohydrodynamic technology. J. Seismol..

[11] Neeshpapa A., Antonov A., Agafonov V. (2014). A low-noise DC seismic accelerometer based on a combination of MET/MEMS sensors. Sensors.

[12] Kozlov V.A., Agafonov V.M., Bindler J., Vishnyakov A.V. (2006). Small, low-power, low-cost IMU for personal navigation and stabilization systems. Proc. Inst. Navig. Natl. Tech. Meet..

[13] Zaitsev D., Agafonov V., Egorov E.V., Antonov A.N., Krishtop V. (2016). Precession Azimuth Sensing with Low-Noise Molecular Electronics Angular Sensors. Sensors.

[14] Kapustian N., Antonovskaya G., Agafonov V., Neumoin K., Safonov M. (2013). Seismic Monitoring of Linear and Rotational Oscillations of the Multistory Buildings in Moscow. Seism. Behav. Des. Irregul. Complex. Civil. Struct..

[15] Agafonov V.M. (2018). Modeling the Convective Noise in an Electrochemical Motion Transducer. Int. J. Electrochem. Sci..

[16] Sun Z., Agafonov V.M. (2010). 3D numerical simulation of the pressure-driven flow in a four-electrode rectangular micro-electrochemical accelerometer. Sens. Actuat. B-Chem..

[17] Agafonov V.M., Nesterov A.S. (2005). Convective Current in a Four-Electrode Electrochemical Cell at Various Boundary Conditions at Anodes. Russ. J. Electrochem..

[18] Agafonov V.M., Krishtop V.G. (2004). Diffusion Sensor of Mechanical Signals: Frequency Response at High Frequencies. Russ. J. Electrochem..

[19] Kozlov V.A., TerentEv D.A. (2003). Transfer Function of a Diffusion Transducer at Frequencies Exceeding the Thermodynamic Frequency. Russ. J. Electrochem..

[20] Aseev G.G. (1998). Electrolytes Transport Phenomena: Methods for Calculation of Multicomponent Solutions and Experimental Data on Viscosities and Diffusion Coefficients.

[21] Anikin E.A., Egorov E.V., Agafonov V.M. (2018). Dependence of self-noise of the angular motion sensor based on the technology of molecular-electronic transfer, on the area of the electrodes. IEEE Sens. Lett..

